# Bio-Control of *Anopheles* Mosquito Larvae Using Invertebrate Predators to Support Human Health Programs in Ethiopia

**DOI:** 10.3390/ijerph18041810

**Published:** 2021-02-12

**Authors:** Kasahun Eba, Luc Duchateau, Beekam Kebede Olkeba, Pieter Boets, Dechasa Bedada, Peter L. M. Goethals, Seid Tiku Mereta, Delenasaw Yewhalaw

**Affiliations:** 1 Biometrics Research Centre, Faculty of Veterinary Medicine, Ghent University, 9820 Merelbeke, Belgium; luc.duchateau@ugent.be; 2 Department of Environmental Health Science and Technology, Jimma University, Jimma 378, Ethiopia; beekamkebede@gmail.com (B.K.O.); seidtiku@yahoo.com (S.T.M.); 3 Department of Animal Sciences and Aquatic Ecology, Ghent University, Coupure Links 653, Building F, 9000 Ghent, Belgium; pieter.boets@oost-vlaanderen.be (P.B.); peter.goethals@ugent.be (P.L.M.G.); 4 Department of Environmental Health Science, Hawassa University, Hawassa 1560, Ethiopia; 5 Provincial Centre of Environmental Research, Godshuizenlaan 95, 9000 Ghent, Belgium; 6 Department of Statistics, Jimma University, Jimma 378, Ethiopia; decheebe@gmail.com; 7 School of Medical Laboratory Sciences, Jimma University, Jimma 378, Ethiopia; delenasawye@yahoo.com; 8 Tropical and Infectious Diseases Research Center, Jimma University, Jimma 378, Ethiopia

**Keywords:** malaria, *Anopheles* mosquito, bio-control, predation efficacy, Ethiopia

## Abstract

Mosquitoes have been a nuisance and health threat to humans for centuries due to their ability to transmit different infectious diseases. Biological control methods have emerged as an alternative or complementary approach to contain vector populations in light of the current spread of insecticide resistance in mosquitoes. Thus, this study aimed to evaluate the predation efficacy of selected potential predators against *Anopheles* mosquito larvae. Potential invertebrate predators and *Anopheles* larvae were collected from natural habitats, mainly (temporary) wetlands and ponds in southwest Ethiopia and experiments were conducted under laboratory conditions. Optimal predation conditions with respect to larval instar, water volume and number of predators were determined for each of the seven studied predators. Data analyses were carried out using the Poisson regression model using one way ANOVA at the 5% significant level. The backswimmer (Notonectidae) was the most aggressive predator on *Anopheles* mosquito larvae with a daily mean predation of 71.5 larvae (95% CI: [65.04;78.59]). Our study shows that larval instar, water volume and number of predators have a significant effect on each predator, except for dragonflies (Libellulidae), with regard to the preference of the larval instar. A selection of mosquito predators has the potential to control *Anopheles* mosquito larvae, suggesting that they can be used as complementary approach in an integrated malaria vector control strategy.

## 1. Background

Mosquitoes are recognized as major vectors of human diseases transmitting malaria, lymphatic filariasis, yellow fever, dengue and others [1]. Mosquito-borne diseases remain a major problem in almost all tropical and subtropical countries. The Ethiopian National Malaria Indicator Survey 2015 revealed that 65% of the districts in the country were malarious, and 53% had a risk of moderate to high transmission [2]. Ethiopia has been implementing a phase III national malaria strategic plan aiming to meet the ambitious goal of partially eliminating malaria in 50 districts by 2020 and entirely by 2030 [3].

Environmental management is implemented in the control of mosquito populations along with chemical or microbiological methods in different parts of the world, especially where mosquito-borne diseases are endemic [4]. However, mosquito control programs are facing important and timely challenges, including the recent outbreak of arboviral diseases, the development of resistance in several mosquito species and the rapid spreading of highly invasive mosquitoes worldwide [5]. Mosquitoes are becoming increasingly resistant to chemical insecticides and there is growing concern about the potential health and environmental risks of these products [6]. The current status of insecticide resistance in mosquitoes [7,8], the effects of insecticides on nontarget insect species [9,10] and the fact that they remain in the environment for decades [11,12,13] are major concerns. Some chemical insecticides also kill nontarget species including mosquito predators, thereby increasing the occurrence of mosquito vectors as mosquitoes could re-establish their population faster than predators after the application of insecticides [14], whereas predators usually have longer life cycles than their prey [15]. In addition, predators are late colonizers of a given habitat after certain disturbances including insecticide application. In Ethiopia, multiple insecticide resistance coupled with the occurrence of high knockdown resistance frequency in *An. arabiensis* populations was reported [16], compelling scientists to evaluate alternative tools to control malaria and other disease vectors. Biological and/or environmental management methods can be used to reduce mosquito vector populations without affecting the environment [17]. Moreover, utilizing biological organisms to control mosquito larvae is not only eco-friendly, but constitutes a means by which more effective and sustainable control can be achieved [18].

Biological control is often an overlooked approach for vector control [19]. On the other hand, there are reports that biological control methods have emerged as the best alternatives to synthetic insecticides and offer great promise in containing vector populations below threshold levels [20]. Moreover, in the context of environmental crisis and global changes, environmentally friendly methods should be encouraged [21]. Mosquito larvae and their predators coexist in a variety of aquatic habitats, ranging from small and temporary aquatic habitats to large and permanent sites. Different macroinvertebrate predators in the orders Odonata, Coleoptera, Diptera and Hemiptera have been shown to be potential biological control agents against mosquito species [22,23]. Furthermore, fishes have been extensively studied both in the laboratory and in the field for their ability to predate mosquito larvae and their use as mosquito biological control agents [1]. It was also reported that Hemiptera, Odonata and Diptera predators are cosmopolitan and promising for biological control [18]. Predation on the larval stages of the mosquitoes with a preference for late instar prey, could reduce the mosquito population density dramatically [24].

A study in Ethiopia by Mereta et al. [25] showed that *Anopheles* larvae occurred less frequently and at lower abundance in habitats where a wide diversity of predators and competitors occurred. Hence, knowledge of predator–prey relationships is pivotal in order to identify mosquito population trends as the density of mosquitoes is very much affected by predation [26]. Evaluation of larval predation efficacy of predators has implications for implementing biological control interventions against mosquitoes. Yet, there is very little evidence for the effectiveness of locally available aquatic macroinvertebrate potential predators on mosquito larvae in Ethiopia. Hence, this study was carried out to evaluate the predation efficacy of selected potential predators against *Anopheles* larvae.

## 2. Materials and Methods

### 2.1. Predators Sampling and Identification

The aquatic macroinvertebrates identified as potential predators in the experiments were collected from natural habitats situated in the Gilgel Gibe watershed, southwest Ethiopia. Collection was made using a scoop net (mesh size of 250 μm) supported by a metal frame. Collected potential predators were transferred to plastic basins with water from the same habitat and transported to the laboratory of the Department of Environmental Health Sciences and Technology, Jimma University. In the laboratory, the potential predators were identified at the family level, based on their morphologies, using an identification key [27]. Afterwards, predators identified at the family level were used in the predation efficacy evaluation experiment. All the potential predators were starved for a period of 12 h during illuminated conditions and subjected to acclimatize the laboratory environment [28]. The experiment was carried out under a normal light cycle. That is, predators were starved for 12 h and experiment was set up at 12:00 pm for the dark phase. The same predators were used for the illuminated phase.

### 2.2. Anopheles Mosquito Larvae Collection

Mosquito larvae were collected by dipping from natural mosquito breeding sites located in the Gilgel Gibe watershed, southwest Ethiopia. During collections, larvae were placed in plastic containers half filled with water from the same breeding site and transported to the laboratory of Department of Environmental Health Sciences and Technology, Jimma University. In the laboratory, all *Anopheles* larva were sorted according to developmental instars (1st, 2nd, 3rd and 4th) based on head capsule sizes [29]. Larvae were provided with dog biscuits to forage until the experiment was started [30].

### 2.3. Study Design

In the first phase of the research, the optimal predation conditions with respect to larval instar, water volume and number of predators were determined for each of the seven different predator families—namely, Aeshinidae, Belostomatidae, Corixidae, Dytiscidae, Gomphidae, Libellulidae and Notonectidae. First, we determined which mosquito larval instar was consumed the most by keeping the volume of water constant (2 L) and introducing 5 predators. Second, 5 predators were fed with the chosen larval instar with water volumes of 1, 2 or 3 L. Finally, the experiment was run by changing the number of predators between 1, 5, 10 and 15, keeping the two other factors at their optimal levels—i.e., largest predation. In each experiment, 50 *Anopheles* larvae were introduced into the experimental buckets. To maintain the prey density, the number of consumed larvae was replenished after 12 h predation—i.e., if 30 larvae were consumed in the first 12 h, 30 larvae were added to maintain the maximum larvae to 100. The development of the mosquito larvae to the next instar was monitored every 12 h and when there was development to next instar, the larvae were removed and replaced in each experiment and control groups.

In the second phase, predation was compared between illuminated (6:00 a.m.–6:00 p.m.) and dark (6:00 p.m.–6:00 a.m.) conditions for each predator family, using the optimal conditions obtained in the first phase. In the third phase, predation was compared between the predators during a 24 h period again using the optimal conditions recorded in the first phase. In all three phases of the experiment, the same numbers of *Anopheles* mosquito larvae were added in each experimental and control group without predators to monitor larval mortality.

**Figure 1:**
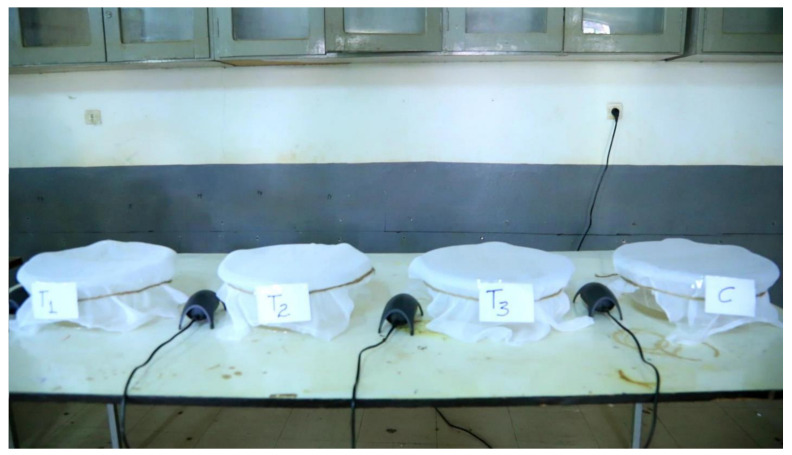
Schematic diagram of experimental set up.

The water used for both the experiment and control groups was collected from natural habitats of mosquitoes and checked for larvae to avoid carrying over before the start of the experiment. Dog biscuit was added as feed for larvae in all experimental and control groups. Each bucket was properly covered with nylon mesh in order to prevent colonization of the water by any insect or other organisms and to avoid oviposition by mosquitoes. The buckets used for this experiment contained a similar volume of water as that encountered in the natural breeding habitats of the principal vector of malaria (*An. arabiensis*) in Ethiopia which typically breeds in small to medium breeding habitats. Additionally, the predators had space to fly or move around as in their natural breeding habitats (Figure 1). To sustain the dissolved oxygen, the treatment and control groups were aerated using an aerator (Tetra APS 50 Aquarium Air Pumps, Swell UK Ltd, Cheshire, UK). Experimental treatments and controls were randomly assigned. In this study, there were seven treatments, each treatment with three replicates and one control.

### 2.4. Data Analysis

All analyses were based on the Poisson regression model using Chi-squared tests at the 5% significance level (SAS version 9.4, SAS Institute Inc., Cary, NC, USA) Mean separation for significant ANOVA was carried out using Tukey’s student zed range test (honestly significant difference (HSD)). The *p*-value was adjusted for multiple comparisons using Tukey’s adjustment technique.

## 3. Results

### 3.1. Optimum Predation Conditions

In this study, the predation experiments revealed that all predators evaluated consumed *Anopheles* mosquito larvae. The optimal predation condition with respect to larval instar and water volume varies among predator families except for the number of predators where the highest number (n = 15) is optimal for all predator families. The optimal condition for the Notonectids, the most efficient predator, is first instar larvae and 1 L volume of water, whereas fourth instar larvae and 3 L volume of water was found to be the optimal condition for Dytiscidae (Table 1).

### 3.2. Predation Efficiency under Illuminated and Dark Conditions

**Figure 2:**
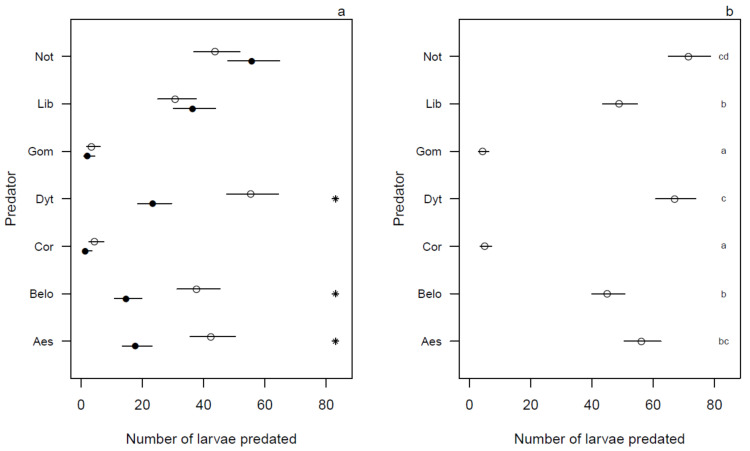
Mean mosquito larval predation (with bars representing the 95% confidence interval) for each predator (Not = Notonectidae, Dyt = Dytiscidae, Aes = Aeshinidae, Lib = Libellulidae, Bel = Belostomatidae, Cor = Corixidae, Gom = Gomphidae), with panel a comparing the illuminated and dark period for each predator, and panel b comparing the predators over a 24 h period. * corresponds to a significant difference between the illuminated and dark periods in panel (**a**). Means within a column followed by the same letter(s) are not significantly different in panel (**b**). Mean separation for significant ANOVA was carried out using Tukey’s student zed range test.

Larval predation of Belostomatidae, Aeshinidae and Dytiscidae was significantly higher under illuminated compared to dark conditions (*p* < 0.001) (Figure 2a), with no significant differences for the other predators.

### 3.3. Predation Efficiency during 24 h

Notonectidae was the most aggressive predator against *Anopheles* mosquito larvae with a daily mean predation of 71.5 larvae (95% CI: [65.04;78.59]). The second most efficient predator was Dytiscidae, with a daily mean predation of 67 larvae (95% CI: [60.76;73.88]). On the other hand, minimum mean daily larval predation was recorded for Corixidae and Gomphidae (Figure 2b).

## 4. Discussion

The findings of this study demonstrated that all evaluated predators consumed different larval instars of *Anopheles* mosquito, but with varying predation rates depending on the taxon and the conditions such as water volume and number of predators. Similarly, previous studies documented that macroinvertebrate taxa belonging to the Hemipterans, Odonates and Coleopterans have been thought to be important potential mosquito larvae predators [18,31,32,33,34,35,36,37].

Our study demonstrated that Notonectids are the most aggressive predator against the *Anopheles* mosquito larvae followed by Dytiscidae, Aeshinidae, Libellulidae, Belostomatidae, Corixidae and Gomphidae, consecutively. Our observation corroborates earlier observations [18,32,33,35,38], which demonstrated promising predation efficiency of notonectids on mosquito larvae. Notonectids were able to completely clear tubs from all contained mosquito larvae in semifield experiments [39,40]. This might be due to the ability of Notonectids to swiftly dive under water (submerged predator) and frequently come to the surface for breathing which makes it an efficient predator for *Anopheles* larvae and making the mosquito larvae a preferred prey [41]. Notonectids are also capable of aerial dispersal between ephemeral aquatic habitats of varied volumes. Given that the conditions are adequate for the notonectids to establish themselves, their promotion in aquatic systems could thus help reduce the proliferation of mosquito malaria vectors [42]. In addition, previous studies showed that in addition to Notonectidae, Dytiscidae [1,22,43], Belostomatidae [34,44], Aeshinidae [45] and Libellulidae [41] were all voracious predators of mosquito larvae.

In our study, larval predation of Belostomatidae, Aeshinidae and Dytiscidae was significantly higher under illuminated compared to dark conditions which was demonstrated before for other predators [1] and specifically for Dytiscidae [46].

The results of our study indicated that mosquito larval instars, volumes of water and number of predators were factors affecting the predation efficacy of all predators except for Libellulidae with regard to preference of the larval instars. Our findings are in line with the results of previous studies [9,18,38,47,48] which reported that mosquito larval consumption by predators depends on mosquito larval instar. The mean larval predation of the most aggressive predator, notonectids, was highest at 1 L volume of water. A previous study also documented that the number of consumed larvae decreased with increasing search area or water volume, and the highest predation was observed at 1 L water volume [49,50]. Increasing the volume of water had a negative effect on the predation rate, perhaps due to the evasion tactics of the mosquito larvae [23,51,52].

The findings presented here on evaluation of predation efficacy of potential predators against *Anopheles* mosquito larvae have implications for designing bio-control tools as a complementary vector control strategy or as component of an integrated vector control program in Ethiopia. The bio-control could be especially effective during the dry season for localities where breeding sites are few, fixed and discoverable. Currently, the control of adult mosquitos is difficult because of widespread insecticide resistance. Hence, the use of potential aquatic predators could be an alternative or complementary control measure for reduction in the adult mosquito population to reduce malaria transmission in Ethiopia and elsewhere in regions with similar eco-epidemiological settings. However, prior to incursion of the efficient predators as a bio-control of *Anopheles* mosquito larvae, it is important to understand their ecological impact. Rearing and introducing predators to natural mosquito breeding habitats could be used as a bio-control method to control immature *Anopheles* mosquitoes. However, the introduction of taxa for bio-control could result in negative impacts on ecosystems if their role in the food web was not carefully evaluated. The substantial predatory impacts of notonectids towards *Anopheles* mosquito larva prey, especially at the early stages of larvae and at higher density in small pools, could assist the bio-control of *Anopheles* mosquito larvae.

## 5. Conclusions

The results of the present study demonstrated that all evaluated predators in this experiment consumed *Anopheles* mosquito larvae. Notonectidae was the most efficient predator, while Corixidae was the least efficient predator. Water volume, mosquito larval instars and numbers of predators were important factors in determining the predation efficacy. Overall, the predation efficacy of the evaluated predators was high, suggesting the use of these predators as bio-control tools either alone or as a component of integrated vector management in the control of mosquito vectors. Moreover, the findings of this study can be used as baseline for further investigation on screening the most efficient species of the predators and whether they prefer to prey on *Anopheles* mosquito larvae.

## Figures and Tables

**Table 1:** The optimal conditions for larval predation in terms of larval instar, water volume and number of predators and the number of larvae consumed (95% confidence interval) at that optimal level for the 7 different predators.

Predator Family	Mosquito Instar	Water Volume	# of Predators	# of Larvae Consumed
Aeshinidae	4	2	15	109 (98;121)
Belostomatidae	2	2	15	90 (80;101)
Corixidae	2	2	15	20 (16;26)
Dytiscidae	4	3	15	168 (154;184)
Gomphidae	3	3	15	33 (27;40)
Libellulidae	4	2	15	29 (24;36)
Notonectidae	1	1	15	140 (127;154)

# = number.

## Data Availability

Data supporting the conclusions of this article are included within the article. The dataset generated and/or analysed during the present study is available from the corresponding author.
